# Tracheotomy in the intensive care unit: guidelines from a French expert panel

**DOI:** 10.1186/s13613-018-0381-y

**Published:** 2018-03-15

**Authors:** Jean Louis Trouillet, Olivier Collange, Fouad Belafia, François Blot, Gilles Capellier, Eric Cesareo, Jean-Michel Constantin, Alexandre Demoule, Jean-Luc Diehl, Pierre-Grégoire Guinot, Franck Jegoux, Erwan L’Her, Charles-Edouard Luyt, Yazine Mahjoub, Julien Mayaux, Hervé Quintard, François Ravat, Sebastien Vergez, Julien Amour, Max Guillot

**Affiliations:** 1 Service de Réanimation, Groupe Hospitalier Pitié-Salpêtrière, Assistance Publique-Hôpitaux de Paris, Paris, France; 2 Hôpitaux Universitaires de Strasbourg, Nouvel Hôpital Civil, Pôle d’Anesthésie-Réanimation Chirurgicale, SAMU, SMUR, NHC, 1 Place de l’Hôpital, 67000 Strasbourg, France; 3 EA 3072, FMTS, Université de Strasbourg, Strasbourg, France; 4 Intensive Care Unit and Department of Anesthesiology, Research Unit INSERM U1046, University of Montpellier Saint Eloi Hospital and Montpellier School of Medicine, Montpellier, France; 5 Medical-Surgical Intensive Care Unit, Gustave Roussy Cancer Campus, Villejuif, France; 6 CHRU Besançon 25000, EA3920 Université de Franche-Comté, Besançon, France; 7 Australian and New Zealand Intensive Care Research Centre, Department of Epidemiology and Preventive Medicine, Monash University, Clayton, Australia; 8 SAMU de Lyon and Department of Emergency Medicine, Hospices Civils de Lyon, Edouard Herriot Hospital, Lyon, France; 9 Lyon Sud School of Medicine, University Lyon 1, Oullins, France; 10 Department of Preoperative Medicine, University Hospital of Clermont-Ferrand, Clermont-Ferrand, France; 11 R2D2, EA-7281, Auvergne University, Clermont-Ferrand, France; 12 INSERM, UMRS1158 Neurophysiologie Respiratoire Expérimentale et Clinique; AP-HP, Groupe Hospitalier Pitié-Salpêtrière Charles Foix, Service de Pneumologie et Réanimation Médicale du Département R3S, Sorbonne Université, Paris, France; 13 Medical ICU, AP-HP, Georges Pompidou European Hospital, Paris, France; 14 INSERM UMR-S1140, Paris Descartes University and Sorbonne Paris Cité, Paris, France; 15 Anaesthesiology and Critical Care Department, Amiens University Hospital, Place Victor Pauchet, 80054 Amiens, France; 16 INSERM U1088, Jules Verne University of Picardy, 80054 Amiens, France; 17 Service ORL et Chirurgie Cervico-maxillo-Faciale, CHU PONTCHAILLOU, Rue H. Le Guilloux, 35033 Rennes Cedex 9, France; 18 CeSim/LaTIM INSERM UMR 1101, Université de Bretagne Occidentale, Rue Camille Desmoulins, 29200 Brest Cedex, France; 19 Médecine Intensive et Réanimation, CHRU de Brest, Boulevard Tanguy Prigent, 29200 Brest Cedex, France; 20 UPMC Université Paris 06, INSERM, UMRS-1166, ICAN Institute of Cardiometabolism and Nutrition, Sorbonne Universités, Paris, France; 21 Department of Anesthesia and Intensive Care, Amiens-Picardie University Hospital, Amiens, France; 22 Réanimation médico chirurgicale Hôpital Pasteur 2 CHU de Nice, 30 voie romaine, 06000 Nice, France; 23 CNRS UMR 7275, IPMC Sophia Antipolis, Valbonne, France; 24 Centre des brûlés, Centre Hospitalier St Joseph et St Luc, 20 quai Claude Bernard, 69007 Lyon, France; 25 ORL Chirurgie Cervicofaciale, CHU Toulouse Rangueil-Larrey, 24 chemin de Pouvourville, 31059 Toulouse Cedex 9, France; 26 Département d’Anesthésie et de Réanimation Chirurgicale, Institut de Cardiologie, Groupe Hospitalier Pitié-Salpêtrière, 47-83 Boulevard de l’Hôpital, 75013 Paris, France; 27 Hôpitaux Universitaires de Strasbourg, Hôpital de Hautepierre, Réanimation Médicale, Avenue Molière, 67200 Strasbourg, France

## Abstract

Tracheotomy is widely used in intensive care units, albeit with great disparities between medical teams in terms of frequency and modality. Indications and techniques are, however, associated with variable levels of evidence based on inhomogeneous or even contradictory literature. Our aim was to conduct a systematic analysis of the published data in order to provide guidelines. We present herein recommendations for the use of tracheotomy in adult critically ill patients developed using the Grading of Recommendations Assessment, Development, and Evaluation (GRADE) method. These guidelines were conducted by a group of experts from the French Intensive Care Society (Société de Réanimation de Langue Française) and the French Society of Anesthesia and Intensive Care Medicine (Société Francaise d’Anesthésie Réanimation) with the participation of the French Emergency Medicine Association (Société Française de Médecine d’Urgence), the French Society of Otorhinolaryngology. Sixteen experts and two coordinators agreed to consider questions concerning tracheotomy and its practical implementation. Five topics were defined: indications and contraindications for tracheotomy in intensive care, tracheotomy techniques in intensive care, modalities of tracheotomy in intensive care, management of patients undergoing tracheotomy in intensive care, and decannulation in intensive care. The summary made by the experts and the application of GRADE methodology led to the drawing up of 8 formal guidelines, 10 recommendations, and 3 treatment protocols. Among the 8 formal guidelines, 2 have a high level of proof (Grade 1+/−) and 6 a low level of proof (Grade 2+/−). For the 10 recommendations, GRADE methodology was not applicable and instead 10 expert opinions were produced.

## Background

Tracheotomy is a procedure commonly used in intensive care, albeit with great disparities between medical teams in terms of frequency (5–54%) and modality (surgical or percutaneous) [1, 2]. Although tracheotomy has a long history, its utility, indications, duration, and techniques are the subject of debate [3, 4]. Also, the real or potential advantages of tracheotomy need to be weighed against its risks, which are rare but sometimes serious. The advantages are a reduction in pharyngolaryngeal lesions, lower risk of sinusitis, reduced sedation requirements, easier buccopharyngeal hygiene, improved patient comfort with easier communication, facilitated care by nursing personnel, maintenance of swallowing, possible glottic closure, simpler reinsertion in cases of accidental decannulation, and easier weaning from mechanical ventilation [5]. In some studies, early use of tracheotomy was associated with decreased incidence of ventilator-acquired pneumonia, reduced duration of mechanical ventilation and of intensive care, and so of costs, and decreased hospital mortality [6, 7]. However, several recent randomized trials found no evidence of these benefits [8–11]. The most frequent complications can be qualified as minor (for example, minor stomal bleeding). Rare and life-threatening complications, such as lesions of the brachiocephalic artery trunk, have been reported.

Among the controversies surrounding tracheotomy in intensive care, the greatest is probably that of its indication. Tracheotomy is most often considered in cases of failed extubation and of prolonged mechanical ventilation. Three remarks are relevant here. First, there is currently no consensus regarding the contribution of failed extubation (one, two, three attempts? in what conditions?) and of prolonged mechanical ventilation. Second, it may be worthwhile preventing failure of extubation and not adding the deleterious effects of prolonged intubation to those of tracheotomy. The intensivist should predict the failure of extubation and the duration of ventilation so as to perform tracheotomy without delay [5], but prediction of the duration of ventilation is an inexact “science” [12, 13]. Third, the duration of mechanical ventilation and the success of extubation depend on intensive care management as a whole (notably the appropriate treatment of an infection, the water–sodium balance and acid–base balance, nutrition, and sedation). In particular, a sedation protocol is essential.

The most recent SRLF guidelines concerning the surgical approach to the trachea of ventilated patients in intensive care date back to 1998 [14]. There are no recent international guidelines and national guidelines are rare [15, 16]. In the absence of clearly defined and unquestionable criteria, tracheotomy is most often decided solely by the medical team in charge of the patient. In the last ten or so years, the medical literature has been enriched by new clinical data, often compiled in the form of meta-analyses [17–19]. It was against this backdrop that the Société de Réanimation de Langue Française (SRLF) and the Société Française d’Anesthésie et de Réanimation (SFAR) decided to draw up the present guidelines entitled “Tracheotomy in the Intensive Care Unit.” The aim of these guidelines is to define the indications, contraindications, modalities, and monitoring of tracheotomy in light of the current literature data.

## Methods

These guidelines were prepared by a working group of experts from the SRLF and the SFAR. The organizing committee, together with the coordinators, first defined the questions to be addressed and then designated the experts in charge of each question. The questions were formulated according to the Patient Intervention Comparison Outcome (PICO) format. Grade of Recommendation Assessment, Development and Evaluation (GRADE) methodology was used to analyze the literature and formulate guidelines. A level of proof was defined for each bibliographical reference cited, as a function of the type of study. This level of proof could be reviewed in light of the methodological quality of the study. An overall level of proof was determined for each endpoint, taking into account the level of proof of each reference, the between-study consistency of the results, the direct or indirect nature of the proof, and cost analysis. A “strong” overall level of proof enabled formulation of a “strong” guideline (must be done, must not be done… GRADE 1 + or 1 −). A “moderate,” “weak,” or “very weak” overall level of proof led to the writing of an “optional” guideline (should probably be done or should probably not be done… GRADE 2 + or 2 −). When the literature was inexistent, the question could be the subject of a guideline in the form of an expert opinion (the experts suggest…). The proposed guidelines were presented and discussed one by one. The aim was not necessarily to reach a single, unanimous opinion of all the experts for each proposal, but to derive points of agreement or disagreement and of indecision. Each expert then reviewed every guideline and rated it using a scale from 1 (complete disagreement) to 9 (complete agreement). The collective rating was done using a GRADE grid. To validate a guideline on a criterion, at least 50% of the experts had to be in broad agreement, while < 20% of them expressed the opposite opinion. For a guideline to be strong, at least 70% of the experts had to be in broad agreement. In the absence of strong agreement, the guidelines were reformulated and again rated, with a view to reaching a consensus.

### Topics of the guidelines: summary of the results

Because of the specificity of emergency airway management (in emergency medicine or intensive care, and in particular in patients with cervicofacial trauma or burns), we did not include it in our literature analysis or in the guidelines. We shall, therefore, address tracheotomy only in the setting of planned tracheotomy in adults in intensive care.

Five topics were defined: indications and contraindications for tracheotomy in intensive care, tracheotomy techniques in intensive care, modalities of tracheotomy in intensive care, management of patients undergoing tracheotomy in intensive care, and decannulation in intensive care. An extensive search of the bibliography from recent years was performed using PubMed and the Cochrane database. To be selected for the analysis, articles had to be written in English or in French.

The summary made by the experts and the application of GRADE methodology led to the drawing up of 8 formal guidelines, 10 recommendations, and 3 treatment protocols. Among the 8 formal guidelines, 2 have a high level of proof (Grade 1+/−) and 6 a low level of proof (Grade 2+/−). For the 10 recommendations, GRADE methodology was not applicable and instead 10 expert opinions were produced. After 2 rounds of rating and various amendments, strong agreement was obtained for all the guidelines and protocols.

### Indications and contraindications for tracheotomy in intensive care

#### **R1.1**

The experts suggest that tracheotomy be proposed in cases of prolonged weaning from mechanical ventilation and of acquired and potentially reversible neuromuscular disorder.

**(Expert opinion)**

#### **Rationale**

The term neuromuscular refers to acquired and potentially reversible cerebrospinal, motor, and muscle disorders (e.g., Guillain–Barré syndrome, intensive care unit acquired muscle weakness, myasthenia, lupus myelitis). No study has provided formal evidence that tracheotomy improves the prognosis for survival of patients with these types of disorders. In this indication, no randomized study has evaluated the specific usefulness of early compared with late tracheotomy. Nevertheless, studies, often retrospective, suggest that late tracheotomy raises the risk of ventilator-associated pneumonia [20]. Tracheotomy can be proposed when weaning from mechanical ventilation is prolonged: weaning lasting more than 7 days after the first spontaneous breathing trial [21].

In the case of Guillain–Barré syndrome, tracheotomy should only be considered if weaning from invasive mechanical ventilation is not achieved after completion of immunotherapy (intravenous immunoglobulins or plasma exchange). At the end of immunotherapy, deficit in plantar flexion associated with sciatic nerve block was found to be an early predictor of prolonged (> 15 days) invasive mechanical ventilation in 100% of cases [22]. Alone, deficit in plantar flexion at the end of immunotherapy had a positive predictive value of 82% for prolonged mechanical ventilation.

#### **R1.2**

The experts suggest that the indication for tracheotomy in patients with chronic respiratory failure should be the subject of multidisciplinary discussion.

**(Expert opinion)**

#### **Rationale**

The usefulness of intermittent mechanical ventilation in the management of patients with chronic respiratory failure is beyond the scope of these recommendations. When intermittent mechanical ventilation is indicated, a randomized study does not seem necessary before recommending first-line noninvasive ventilation rather than tracheotomy.

Life-threatening decompensation of chronic respiratory failure is generally managed in intensive care. In this setting, certain forms of chronic respiratory failure, notably those resulting from neurological disorders, can be managed using tracheotomy to enable mechanical ventilation and to simplify upper airway management. A 2016 meta-analysis including data from a randomized trial and 25 observational studies suggests that intermittent mechanical ventilation can improve the quality of life of patients with chronic respiratory failure [23]. The meta-analysis considered together patients receiving intermittent noninvasive mechanical ventilation and tracheotomized patients. More specifically, several studies have looked into the usefulness of tracheotomy in amyotrophic lateral sclerosis (ALS). In a 2011 study, an Italian team found that of 60 ALS patients who underwent tracheotomy, 44 (70%) left hospital completely dependent on mechanical ventilation, 17 (28%) were partially dependent, and a single patient was completely weaned from mechanical ventilation. At 1-year follow-up, 13 (22%) patients were still alive and had a quality of life deemed similar to that of ALS patients who did not have a tracheotomy [24].

In this type of situation, the patient and his or her family must be informed that tracheotomy does not alter the prognosis of the causal disease. The usefulness of tracheotomy in improving patient comfort and management following a stay in intensive care must be accurately evaluated, in particular with the patient and the medical team. Facilitation of upper airway management does not necessarily lead to improved comfort; tracheotomy can unduly prolong suffering associated with the underlying illness. In a context of chronic respiratory failure, these ethical considerations must be carefully thought through and discussed with the patient and his or her family before performing a tracheotomy.

#### **R1.3**

Tracheotomy in intensive care should not be performed before the fourth day of mechanical ventilation.

**(GRADE 1+/STRONG agreement)**

#### **Rationale**

The question of the timing of tracheotomy in intensive care is hard to analyze, because: 1) it is necessary beforehand to demonstrate the usefulness of tracheotomy (independently of its timing) and 2) most studies comparing early and late tracheotomy include nontracheotomized patients in the “late” group.

Several good-quality prospective studies relate to “objective” criteria (mortality, incidence of ventilator-associated lung injury, duration of mechanical ventilation and of stay in intensive care). Early tracheotomy (in general before the fourth day of mechanical ventilation) is not associated with decreases in mortality, the incidence of ventilator-associated lung injury, or the duration of mechanical ventilation [8–11, 25, 26]. It does seem to reduce the consumption of hypnotic drugs. Improvement in comfort is not proven, and is insufficiently studied, but seems likely when tracheotomy is done early.

Lastly, early tracheotomy in burn patients with cervicofacial involvement and in patients with cervicofacial trauma more properly comes under the heading of emergency tracheotomy and is not within the scope of these guidelines.

#### **R1.4**

The experts suggest that tracheotomy (percutaneous or surgical) should not be performed in intensive care in situations at high risk of complications.

**(Expert opinion)**

#### **Rationale**

The potentially serious complications are hemorrhage, hypoxemia, and neurological deterioration. Most studies have excluded patients at risk of these complications [6, 9, 10, 25]. Tracheotomy should not, therefore, be performed in intensive care in the following situations:

- Hemodynamic instability.
- Intracranial hypertension (intracranial pressure > 15 mmHg).
- Severe hypoxemia: PaO_2_/FiO_2_ < 100 mmHg, with positive expiratory pressure > 10 cmH_2_O.
- Uncorrected bleeding disorders (platelets < 50 000/mm^3^ and/or international normalized ratio > 1.5 and/or partial thromboplastin time > 2 normal).
- Refusal by the patient and/or family.
- Patient is dying or active treatment is being withdrawn.

### Tracheotomy techniques in intensive care

#### **R2.1**

Percutaneous tracheotomy is the standard method in intensive care patients.

**(GRADE 1+/STRONG agreement)**

#### **Rationale**

Several randomized studies have compared the impact of the technique of tracheotomy (percutaneous or surgical) on the incidence of complications (short-, medium-, and long-term), mortality, and cost [27–36]. The great heterogeneity of endpoints (immediate or delayed, minor or major complications) complicates comparison of studies. To date, neither of the two techniques (percutaneous or surgical) has proven superior in terms of mortality or incidence of major complications (respiratory distress, hemorrhagic shock, tracheal stenosis) [37]. A 2014 meta-analysis including 14 randomized studies suggests that the percutaneous technique is associated with a shorter operative time and a decreased incidence of stoma infection and inflammation [37]. The incidence of other complications does not seem to differ between the two tracheotomy techniques [37]. These results, plus the spread and availability of this technique in intensive care units, mean that percutaneous tracheotomy should whenever possible be preferred to surgical technique.

Whatever the technique used, prior training is needed to perform tracheotomy, which must be done by physicians able to manage any complications or accidents quickly.

#### **R2.2**

The experts suggest that medical and surgical teams should discuss and decide upon the tracheotomy technique to be used when there is a risk of complications.

**(Expert opinion)**

#### **Rationale**

Percutaneous tracheotomy can be made difficult, even impossible, by the patient’s condition. For instance, an unstable cervical spine, an anterior cervical infection, a neck that has been treated (surgery or radiotherapy), difficulty in identifying anatomical landmarks (e.g., obesity, short neck, thyroid hypertrophy), or stiffness of the cervical spine are relative contraindications to percutaneous tracheotomy and prompt instead use of surgical tracheotomy [27]. It is nevertheless difficult to draw up formal guidelines. Indeed, at-risk situations are conventionally exclusion criteria for prospective studies. Observational studies have yielded contradictory results on which technique to prefer in cases of morbid obesity, spinal fracture, or a history of tracheotomy [35, 38–53]. A single randomized prospective study has compared surgical tracheotomy with modified percutaneous tracheotomy or so-called mini-surgical percutaneous dilatational tracheotomy (surgical tracheal access followed by a percutaneous procedure) in at-risk situations (anatomical difficulties, coagulation disorders, hypoxemia, hemodynamic instability). This study found no difference between the two techniques in terms of complications [52].

Such situations should therefore prompt discussions between the medical and surgical teams to decide on what benefit tracheotomy provides and which technique is the most suitable. Percutaneous tracheotomy in these situations can be envisioned by an experienced team with access to the technical means to improve the usual procedure: fiberoptic bronchoscopy, cervical Doppler ultrasound, surgical approach to the tracheal rings, tracheotomy equipment adapted to the anatomical problem (e.g., special tracheotomy kits for obese patients).

#### **R2.3**

Percutaneous dilatational tracheotomy should probably be preferred as the standard method in intensive care patients.

**(GRADE 2+/STRONG agreement)**

#### **Rationale**

Several randomized studies have compared the six techniques of percutaneous tracheotomy: multiple dilator, guide wire dilating forceps, single dilator, rotating dilation, balloon dilation, and translaryngeal tracheotomy. These comparisons have in general been made two-by-two with as principal endpoints the duration of the procedure, failure rate defined by a switch to an alternative technique, the rate of major complications, and the rate of minor complications. These techniques are relatively equivalent, with the exception of translaryngeal tracheotomy, which seems to be associated with a higher rate of failure and of complications, notably major [54, 55]. The single dilator technique is associated with a lower failure rate than rotating dilation [56] and a lower rate of minor complications than balloon dilation or dilation with guide wire dilating forceps [57–59]. When the single dilator technique is compared with all the others, it seems to be associated with a higher success rate (corollary of its more widespread use) [60], but also with a higher rate of minor complications (notably minor bleeding and tracheal ring fractures) [60].

### Conditions necessary for tracheotomy in intensive care

#### **R3.1**

Fiberoptic bronchoscopy should probably be performed before and during percutaneous tracheotomy.

**(GRADE 2+/STRONG agreement)**

#### **Rationale**

Fiberoptic bronchoscopy before tracheotomy is advantageous because it helps locate the point of incision, by transillumination and palpation, and helps position the endotracheal tube correctly, below the vocal cords. Fibroscopy directly visualizes all stages of the procedure (incision, placement of the guide wire and of the dilator, dilation) and the position of the tracheotomy tube [61]. Fibroscopy must be available during the tracheotomy and the clinician must be trained.

Three nonrandomized studies seem to suggest that fiberoptic bronchoscopy could be nonsignificantly associated with more complications [62–64], but they are subject to substantial methodological bias and their results seem difficult to interpret.

A single randomized trial in 60 patients has shown that fiberoptic bronchoscopy is associated with a 47% (95% CI 23–64%) decrease in early complications of percutaneous tracheotomy in intensive care [65]. The main complications observed were accidental extubation, perforation of the cuff of the endotracheal tube, and hemorrhage. In addition, the number of incisions needed for tracheotomy was statistically smaller in the fiberoptic bronchoscopy group.

In summary, the only randomized study performed found that there are fewer complications of percutaneous tracheotomy when fiberoptic bronchoscopy is used.

#### **R3.2**

A laryngeal mask airway should probably not be used during percutaneous tracheotomy in intensive care.

**(GRADE 2−/STRONG agreement)**

#### **Rationale**

Several randomized studies have compared two procedures for extubation of the endotracheal tube from the trachea while maintaining invasive mechanical ventilation: extubation followed by placement of a laryngeal mask airway or withdrawal of the endotracheal tube until the cuff is at the level of the vocal cords. A 2014 meta-analysis of 8 randomized controlled trials of the usefulness of placement of a laryngeal mask airway [66] showed that these trials examined four main outcomes: mortality (one study), the proportion of patients with one or more serious adverse events (seven studies), duration of the procedure (six studies), and failure of the procedure requiring a switch to any other procedure (seven studies). For each of these outcomes, the quality of the proof was considered low. Use of a laryngeal mask airway is not associated with decreases in mortality, complication rate, or failure related to the procedure, but does shorten the length of the procedure by an average of 1.46 (1.01–1.92) minutes. A single randomized controlled study conducted after this meta-analysis [67] found that more patients needed conversion to another procedure and had more clinically significant complications with a laryngeal mask airway.

#### **R3.3**

Cervical ultrasound should probably be performed with percutaneous tracheotomy in intensive care.

**(GRADE 2+/Strong agreement)**

#### **Rationale**

Ultrasound visualizes the trachea and the tracheal rings, thus optimizing positioning of point of incision while avoiding injury to blood vessels and/or the thyroid [68]. Four open randomized studies in a total of 560 patients have tested the usefulness of Doppler ultrasound in preventing complications of percutaneous tracheotomy [69–72]. Of 275 patients who underwent ultrasound-guided localization before tracheotomy, 40 (14.5%) presented a complication during or after the procedure. In the absence of Doppler ultrasound, 74 (26%) of the 285 patients presented at least one complication during or after the procedure, i.e., a 44% (95% CI 21–60) decrease in the risk of complications. The risk of puncturing a blood vessel is reduced by localization beforehand. The success of the procedure at the first attempt is significantly greater with Doppler ultrasound: 94.9% (168/177) versus 90.4% (160/177). There is, however, great heterogeneity between these studies, as the randomization procedure is not always well described [70, 71] and the definition of complications is not uniform.

The strength of the recommendation (2 +) is related to the as-yet infrequent use of ultrasound with tracheotomy and to the quality of the randomized trials.

In conclusion, Doppler ultrasound increases the success rate of tracheotomy and reduces its immediate complications, provided the clinician masters the technique.

#### **R3.4**

The experts suggest that antibiotic prophylaxis should not be prescribed for tracheotomy.

**(Expert opinion)**

#### **Rationale**

Because it opens the trachea, percutaneous tracheotomy can be considered as clean-contaminated surgery. The rate of infection of the operative site ranges between 0 and 33% depending on the study. Most studies comparing percutaneous tracheotomy and surgical tracheotomy indicate a higher rate of infection of the operative site for the surgical procedure. The infection rate for percutaneous tracheotomy is generally between 0 and 4%. In a retrospective study in 297 patients who underwent percutaneous tracheotomy, Hagiya et al. [73] reported a significantly lower rate of infection at the tracheotomy site in patients on antibiotic therapy: 2.36 versus 7.25% (*p* = 0.002). In contrast, there is no randomized study that has assessed the usefulness of antibiotic prophylaxis for tracheotomy. The quality of evidence is therefore very poor. The 2010 SFAR update concerning antibiotic prophylaxis in surgery and interventional medicine advises against antibiotic prophylaxis for tracheotomy (whether surgical or percutaneous is not specified) [74].

#### **R3.5**

The experts suggest that a standardized procedure be implemented in intensive care units that perform percutaneous tracheotomy.

**(Expert opinion)**

#### **Rationale**

**Fig. 1:**
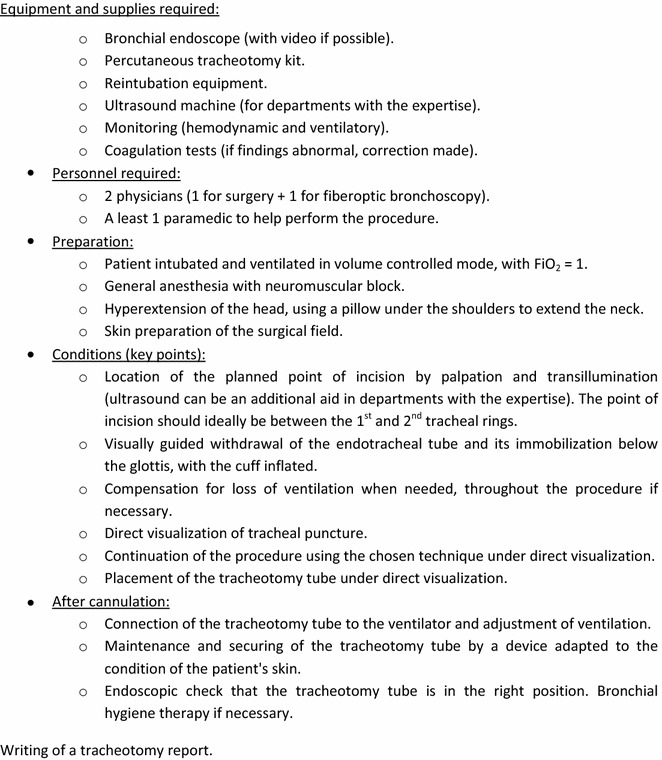
Proposal for a protocol associated with guideline 3.5 (Expert opinion)

**Fig. 2:**
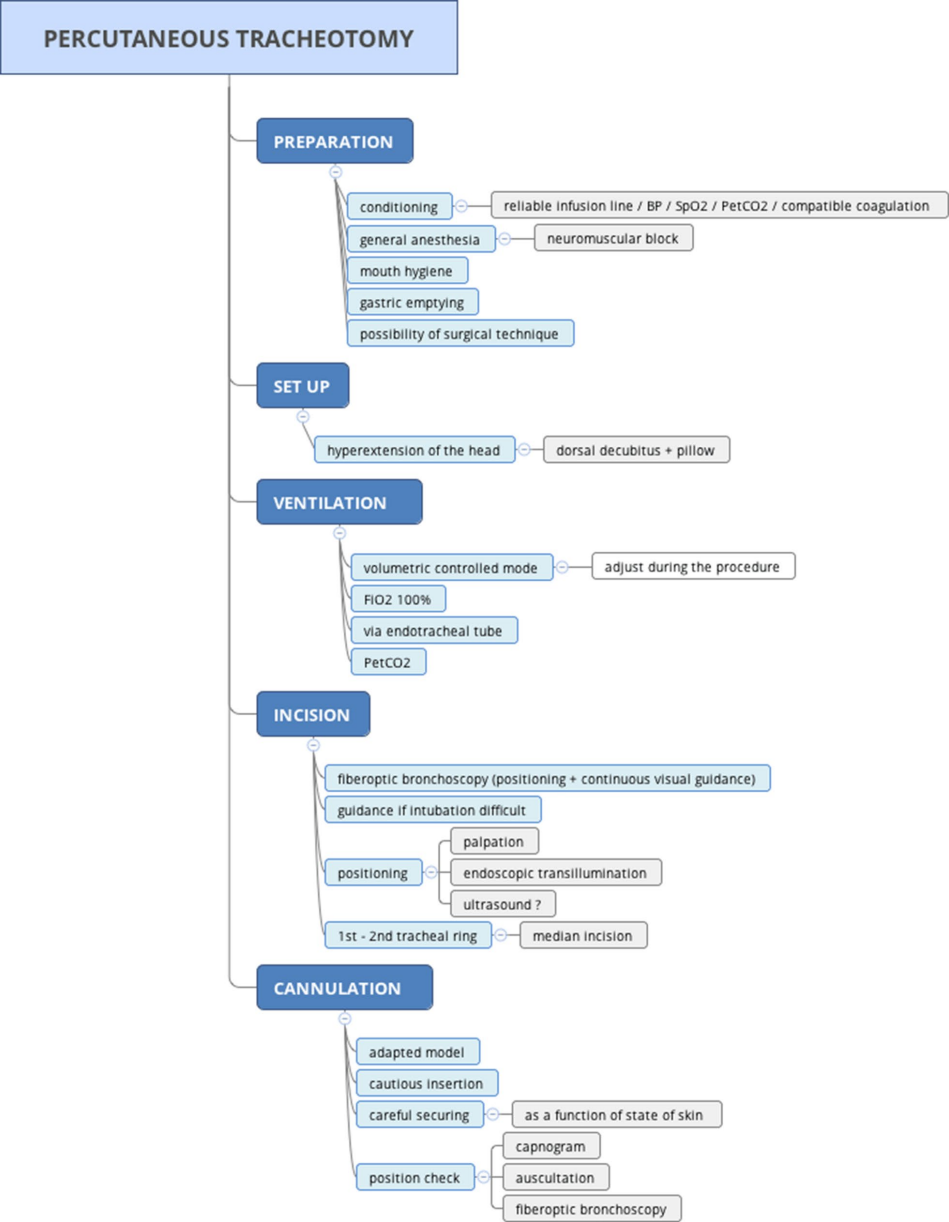
Proposal for a protocol associated with guideline 3.5 (Expert opinion)

Percutaneous tracheotomy in intensive care is an invasive procedure which can lead to potentially serious complications [75] and for which there are contraindications. The learning curve for percutaneous tracheotomy is on average more than 80 consecutive procedures by the same team and with the same technique [76]. In addition, rules should be observed to optimize safety [75]. Intensive care units should define a standard procedure for percutaneous tracheotomy, which could indicate the following points: medical and paramedical personnel required, necessary pre-surgery laboratory tests and radiography, equipment required for airway management, equipment needed for the procedure (notably, the role of Doppler ultrasound and fiberoptic bronchoscopy), position of the patient, method of ventilation, type of analgesia, ways of checking the position of the tracheotomy tube at the end of the procedure, and then the modalities for monitoring the intensive care patient following surgery (Figs. 1, 2).

### Tracheotomy monitoring and maintenance in intensive care

#### **R4.1**

The experts suggest that intensive care units should have a tracheotomy management protocol.

**(Expert opinion)**

#### **Rationale**

The numerous secondary complications of tracheotomy include skin infection, granuloma, secondary bleeding from the stoma, tracheal stenosis, tracheomalacia, and erosion of blood vessels (brachiocephalic vein, brachiocephalic artery) [15, 77, 78]. There is no prospective study comparing different kinds of local care, such as antisepsis, type of dressing, or way of securing. Prospective randomized studies comparing surgical and percutaneous techniques, and different types of percutaneous techniques, do not specify the protocol. Studies evaluating practices for tracheotomy follow-up in intensive care reveal large disparities, absence of formalization, and lack of guidelines for follow-up during or after intensive care [79, 80]. Use of a standard care protocol reduced local lesions [81]. Based on limited data or expert opinions, monitoring is recommended to ensure that cuff pressure does not exceed 30 cmH_2_O [77, 78, 82]. Too low a pressure could lead to inhalation of oropharyngeal secretions [15]. Increased cuff pressure favors ischemia of the tracheal mucosa, which is a source of tracheal stenosis. A check every 8 h is proposed.

Local infection and gastroesophageal reflux damage the cartilage of the tracheal rings, potentially leading to chondritis, tracheal stenosis, and tracheomalacia [83]. By analogy with work done on endotracheal intubation, it is recommended to use tubes fitted with a suction catheter that opens above the cuff, for regular aspiration of retained secretions from the subglottic space.

Special attention should be paid to securing the tracheotomy tube, maintenance of a corrugated tube, and prevention of repeated local trauma caused by the moving and weight of the tubes (avoid pulling the tracheotomy tube). There are no specific data on local care (antisepsis, products, frequency). A single study found no difference in bacterial contamination or local infection between the application of compresses or soft dressings [84]. Few studies specify the performance and type of local care (4–6 applications of isotonic saline, for example, in Lagambina et al.) [77, 85].

The experts consider it useful to check the position of the tracheotomy tube (chest X-ray, ease of tracheal suction, absence of dyspnea) and, if necessary, to use fiberoptic bronchoscopy to look for injury or stenosis, without specifying the frequency or timing.

**Fig. 3:**
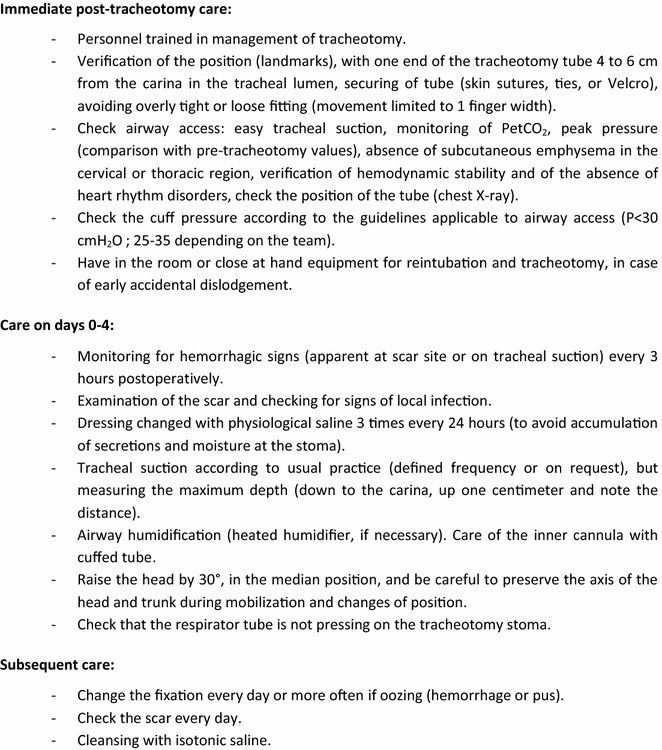
Proposed care protocol associated with guideline 4.1 (Expert opinion)

To meet intensive care safety requirements, management of the tracheotomized patient should include and specify the following: monitoring of the tracheotomy stoma, monitoring of ventilation parameters, specific local care, care of the tracheotomy tube, nature and frequency of the care provided (Fig. 3).

#### **R4.2**

The experts recommend airway humidification in patients with a tracheotomy in intensive care.

**(Expert opinion)**

#### **Rationale**

There are no data on airway humidification in patients with a tracheotomy in intensive care. Lack of airway humidification can lead to obstruction of the tracheotomy tube in patients who need oxygen therapy in intensive care [86]. The UK 2014 guidelines suggest that humidification be envisioned for all patients undergoing tracheotomy. Airway humidification should be adapted in particular to the ventilatory support and to the amount of bronchial secretion [86].

No study has determined which airway humidification technique should be preferred in mechanically ventilated patients undergoing tracheotomy in intensive care. Only two studies have evaluated the effect on the incidence of ventilator-associated lung injury of different humidification systems (heated humidifiers or heat and moisture exchangers) in patients undergoing tracheotomy. Their results are discordant. The first study of 185 patients in each group and only 11 tracheotomized patients [87] found no benefit of airway humidification with any particular system. The second study, in a comparison of only 15 and 16 tracheotomized patients, showed a significant decrease in the incidence of ventilator-associated lung injury in the group with a heated humidifier [88].

#### **R4.3**

The experts suggest that tracheotomy tubes should not be routinely changed in intensive care.

**(Expert opinion)**

#### **Rationale**

No literature study has examined the frequency of tracheotomy tube changes and the incidence of lung disease. A single prospective study in a long-stay hospital for ventilated patients with a tracheotomy showed a reduction in the incidence of granulation tissue when tubes were changed every two weeks [89]. A nonrandomized prospective study in a center for mechanical ventilation weaning showed that a change of tracheotomy tubes before the seventh day after tracheotomy was associated with faster resumption of nutrition and speech. The authors ascribed this effect to a reduction in tracheotomy tube size [90]. They reported no complication associated with the change of tracheotomy tube.

In intensive care, in a practice survey in the USA, 80% of tracheotomy tubes were changed routinely, but with substantial variability [91]. A Dutch practice survey observed that 60% of departments never change the tracheotomy tube [92].

The guidelines of the Belgian Society of Pneumology and the Belgian Association for Cardiothoracic Surgery [15] propose tracheotomy tube changes only if there is a specific indication. The British Intensive Care Society [86] advocates changing a tracheotomy tube without an inner cannula every 7–14 days and a tracheotomy tube with an inner cannula every 30 days. Tube change should be performed no less than 4 days after surgical tracheotomy, and 7–10 days after percutaneous tracheotomy. Subsequently, the frequency of tube change must be adapted to the individual patient’s condition [86].

The European Directive [93] advocates changing medical devices every 30 days. One study shows a structural alteration of the wall of 58% of tracheotomy tubes after 30 days of use [94]. A tracheotomy tube change early in intensive care is associated with risks (tube displacement and respiratory arrest) [15].

In summary, tracheotomy tube change must be guided by clinical considerations and should be envisaged, in particular, in cases of suspected local infection, bleeding, or to reduce the caliber of the tracheotomy tube and to facilitate the patient’s speech.

### Tracheotomy decannulation

#### **R5.1**

The experts suggest that a multidisciplinary decannulation protocol should be available in intensive care units.

**(Expert opinion)**

#### **R5.2**

The tracheotomy tube cuff should probably be deflated when the patient is breathing spontaneously.

**(GRADE 2+/STRONG agreement)**

#### **Rationale**

Numerous observational and before/after studies conclude that use of a weaning protocol shortens weaning time and reduces the decannulation failure rate and the complication rate [95–104]. In a controlled, randomized, single-center trial in 195 patients, cuff deflation once the patient was disconnected from the ventilator reduced failure of decannulation, shortened weaning from mechanical ventilation, and decreased tracheostomy-related complications [105].

**Fig. 4:**
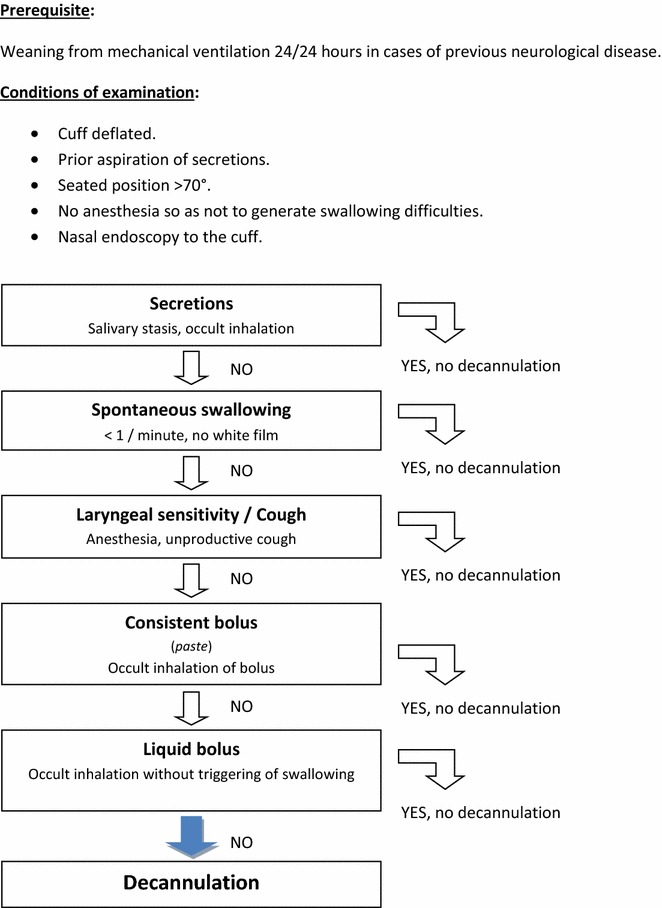
Proposed endoscopic protocol associated with guideline 5.1 (Expert opinion): *(according to Warnecke* et al*. Crit Care Med 2013* (106)*)*

This consensual multidisciplinary protocol, which was written and is applied routinely by all members of the intensive care team who use tracheotomy, should at least define the following (Fig. 4): prior neurological examination and pharyngolaryngeal examinations, medical and paramedical personnel involved in decannulation, equipment needed for decannulation, immediate and subsequent monitoring of decannulation, and type and location of equipment required in cases of respiratory distress following decannulation.

#### **R5.3**

A pharyngolaryngeal examination should probably be performed at or following decannulation.

**(GRADE 2+/STRONG agreement)**

#### **Rationale**

 Few prospective controlled studies consider the pharyngolaryngeal examination required during or following decannulation of intensive care patients or whether or not routine fiberoptic bronchoscopy is needed. A prospective observational study by practitioners blinded to each other’s decisions [106] shows the benefit of routine laryngotracheal endoscopy by the intensivist at decannulation, in comparison with routine clinical assessment of swallowing, possibly completed by the Evans blue dye test. Among the 100 neurological patients in the cohort, endoscopic evaluation allowed successful decannulation in 27 patients for whom clinical assessment had predicted failure of weaning. The recannulation rate was 1.9%. Pharyngolaryngeal examination on decannulation comprises sequential assessments of salivary stasis and silent inhalation, spontaneous swallowing, and laryngeal sensitivity, before considering a swallowing test using paste and then liquid. No patient who passed these three assessments had difficulty swallowing in the tests with paste and liquid.

Other prospective, observational, but noncomparative studies confirm [107, 108]: 1) a higher incidence of swallowing dysfunction in tracheotomized patients ventilated for a prolonged period; 2) a longer intensive care stay and increased risk of inhalation and of pharyngolaryngeal lesions when tracheotomy is prolonged or decannulation is delayed.

This article is being published jointly in Anaesthesia Critical Care & Pain Medicine and Annals of Intensive Care. The manuscript validated by the board of the SRLF (12/13/2016) and the SFAR (12/15/2016).

## Abbreviations

ALS: amyotrophic lateral sclerosis
GRADE: Grading of Recommendations Assessment, Development, and Evaluation
PICO: Patient Intervention Comparison Outcome
SFAR: Société Francaise d’Anesthésie Réanimation
SFMU: Société Française de Médecine d’Urgence
SFORL: Société Française d’Otorhinolaryngologie
SRLF: Société de Réanimation de Langue Française
